# The other face of restriction: modification-dependent enzymes

**DOI:** 10.1093/nar/gkt747

**Published:** 2013-08-29

**Authors:** Wil A. M. Loenen, Elisabeth A. Raleigh

**Affiliations:** ^1^Leiden University Medical Center, P.O. Box 9600 2300RC Leiden, The Netherlands and ^2^New England Biolabs Inc., 240 County Road Ipswich, MA 01938-2723, USA

## Abstract

The 1952 observation of host-induced non-hereditary variation in bacteriophages by Salvador Luria and Mary Human led to the discovery in the 1960s of modifying enzymes that glucosylate hydroxymethylcytosine in T-even phages and of genes encoding corresponding host activities that restrict non-glucosylated phage DNA: *rglA* and *rglB* (restricts glucoseless phage). In the 1980’s, appreciation of the biological scope of these activities was dramatically expanded with the demonstration that plant and animal DNA was also sensitive to restriction in cloning experiments. The *rgl* genes were renamed *mcrA* and *mcrBC* (modified cytosine restriction). The new class of modification-dependent restriction enzymes was named Type IV, as distinct from the familiar modification-blocked Types I–III. A third *Escherichia coli* enzyme, *mrr* (modified DNA rejection and restriction) recognizes both methylcytosine and methyladenine. In recent years, the universe of modification-dependent enzymes has expanded greatly. Technical advances allow use of Type IV enzymes to study epigenetic mechanisms in mammals and plants. Type IV enzymes recognize modified DNA with low sequence selectivity and have emerged many times independently during evolution. Here, we review biochemical and structural data on these proteins, the resurgent interest in Type IV enzymes as tools for epigenetic research and the evolutionary pressures on these systems.

## GENETICS, BIOCHEMISTRY AND STRUCTURES

### Historical sketch

Like conventional modification-blocked restriction, modification-dependent restriction originally was diagnosed owing to its biological effects, when interstrain DNA transfer was unexpectedly inhibited. At the start, phages were the investigatory vehicles, moving between *Escherichia coli* K12, *E. coli* B and *E. coli* C or *Shigella dysenteria* Sh (1,2). Later, reduced plasmid, phage or chromosomal transfer was found when alien modification patterns were present (3–5). Incoming DNA needed the endogenous (for *E. coli* K12) modification of Am6ACN(6)G**T**GC (M.EcoKI; the A opposite the underlined T is also modified) and Gm6ATC (Dam); Cm5CWGG (Dcm) occasionally had effects (6). ‘Outgoing’ DNA was better accepted in many taxa without any of these (7–10).

Progress in cloning and sequencing of restriction enzyme (REase) genes, other nucleases, methyltransferase (MTase) genes and motor proteins began to feed data into efforts to classify sequences and abstract from them signatures predictive of particular functions, e.g. (11–15). Such signatures often correlate with physical protein domains. These domains can be split off from the original protein and added to another and will then operate (mostly) as they are supposed to. This result is the basis for protein tagging with reporters and epitopes by molecular biologists. As we see from the structural organization of modification-dependent REases, this apparently is also the basis for a mix-and-match evolutionary process in real life—grab a DNA-binding domain here, a nuclease domain there, and you’ve got a site-specific (sort of) nuclease! Sometimes, a dimerization surface or a regulatory domain is needed as well.

Finally, with the advent of massive genome sequencing, bioinformatic analysis has become a hypothesis generator so that well-chosen biological and enzymatic tests can (hopefully) allow quick creation of strains and enzymes for further research (16).

### What biological DNA modifications are there?

**Figure 1.**
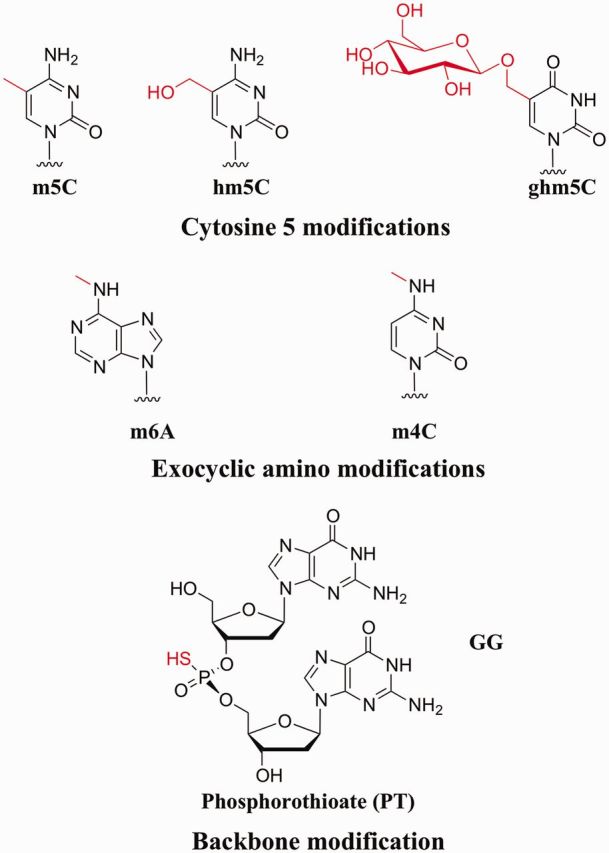
DNA modifications recognized by Type IV enzymes. Enzymatic DNA modifications in the major groove of double-stranded DNA are methylation at cytosine C5 or N4, or at adenosine N6; and glucosylation of a pre-existing 5-hydroxymethylcytosine. The beta-glucosyl derivative is shown; other configurations and other sugars are known to be added by some phages. hm5C is incorporated during replication, after conversion of the dCTP pool to hmdCTP. Phosphorothioate modification of the backbone is carried out postsynthetically. Other biological DNA modifications are known. Only those shown to elicit action of characterized Type IV enzymes are shown here.

Biological DNA modifications have been studied for many years, and much is known about their distribution and the enzymes involved (17–19). Well-known base modifications are C*-*5-methylcytosine (m5C), *N*4-methylcytosine (m4C) and *N*6-methyladenine (m6A) (Figure 1). These are widely distributed in cellular organisms, particularly prokaryotes. Other base modifications have long been known in bacteriophage, prominently 5-hydroxymethylcytosine (hm5C) and derivatives of it with sugar residues attached (ghm5C) (Figure 1), and 5-hydroxymethyluracil (hm5U). Unusual modifications of adenine have also been studied in phage Mu [Mom modification (20)]. Fairly recently, as methods for detection of low frequency modifications have improved, some of these exotic base modifications have also been recognized in higher organisms [hm5C: (21,22)] and lower eukaryotes [hm5U and the sugar-derivatized J base: (23)]. Bioinformatic investigation of coding sequences related to modification enzymes suggests that additional unrecognized base modifications may still be discovered (24,25).

It is not only bases in DNA that may be modified. Enzymatic sulfur modification of the phosphodiester backbone of DNA (PT-DNA, for phosphothioester DNA; Figure 1) has recently been discovered in prokaryotes (26–28). PT-modified DNA is widespread; modification is found in local sequence contexts compatible with sequence-specific addition, and the similarity relationships among the *dnd* genes encoding the modification machinery are consistent with extensive horizontal transfer, as is found for conventional restriction-modification (R-M) systems (29). This opens still further vistas for research on the nature and biological consequences of modification and restriction.

Some of these modifications play important other roles in the life of the host cell, besides restriction wars: in replication timing in prokaryotes and in transcription regulation in prokaryotes and eukaryotes [e.g. (30–33)]. This topic will not be addressed here, except to note that the modifying enzymes that have acquired regulatory effects in bacteria are normally conserved within a clade, unlike cognate-modifying enzymes that accompany R-M systems, which are sporadically distributed (34,35).

### Molecular action: what they do

#### Diversity of modification dependence

Modifications that protect against conventional REases include m5C, hm5C, ghm5C, m6A, m4C and, most recently, PT DNA (with sulfur replacing a non-bridging oxygen). Neither hm5C nor ghm5C are known to be added site-specifically; instead, they are found as universal substitutions in phage DNA. The inverse could also be true: for each protective modification in Figure 1, there are enzymes that attack DNA only when the modification is present (Tables 1 and 2). Many of the enzymes were described only recently and are distinct from the classical examples. Many of the other modifications found in phages (18,19) might be the object of undiscovered Type IV enzymes. hm5U and its glucosylated derivative, J base, and the Mu modification, N6 (1-acetoamido) adenine, would be interesting substrates.

**Table 1.** DNA modifications^a^ that elicit cleavage by Type IV enzymes

Protein	m5C	hm5C	ghm5C	m4C	m6A	PT	References
EcoKMcrA	(+)	(+)	(−)	NT	(−)	NT	(3,36)
ScoA3McrA	+	NT	NT	NT	(+)	+	(37,38); Sco4631
EcoKMcrBC	+	+	−	(+)	−	NT	(3,39,40)
BanUMcrB	(+)						(41)
BanUMcrB3	(+)						(41)
EcoKMrr	(+)	(−)	(−)	(−?)	(+)	NT	(42,43)
BanUMrr	(+)				(+?)		(41)
ScoA3Mrr	(−)				(+)		(37); Sco4213
ZmoMrr	(+?)				(+?)		(44); ZMO1932; Zmrr
SauUSI	+	+	−	−	−?	NT	(45)
SauNewI	(+)						(46); NWMN_2386
SepRPMcrR	(+)						(46); SERP2052
ScoA3I	(+)						(37); Sco2863
PvuRts1I family	+/−	+	+	NT	−	NT	(47,48)
GmrSD	−	−	+	NT	−	NT	(49)
ScoA3II+III	(+)				(−)		(37); Sco3261-62

^a^Modifications: m5C: 5-methylcytosine; hm5C: 5-hydroxymethylcytosine; ghm5C; glucosylated hydroxymethylcytosine; m4C: *N4-*methylcytosine; m6A: *N6-*methyladenine; PT: phosphorothioation of non-bridging oxygen in DNA linkages, also called S-DNA.

+/−: at least 100-fold less activity on this substrate than on substrates with + entry.

(−), (+), based on *in vivo* restriction of phage infection or plasmid transformation with appropriate host mutant configurations; *in vitro* cleavage results have not been reported.

(+?) either m5C or m6A is recognized; these were not distinguished in the reported experiments.

−?: m6A sites tested were not cleaved, but few modified sequences were tested.

NT: not tested.

Where the name found in REBASE (and listed at the left) is not the same as that used in the cited report, the genomic locus_ID is given in the References column, or the name used in the publication.

**Table 2.** DNA modifications^a^ that elicit cleavage by other modification-dependent enzymes (Type IIM)

Protein	m5C	hm5C	ghm5C	m4C	m6A	PT	References
DpnI	−	−	−	−	+	NT	(50)
MspJI family	+	+	−	−	−	NT	(51,52)
SgeI	+						(53)
AoxI	+						
BisI	+						
BlsI	+						
GlaI	+						(54)
GluI	+						
KroI	+						
MalI	+						
MteI	+						
PcsI	+						

^a^Modifications: m5C: 5-methylcytosine; hm5C: 5-hydroxymethylcytosine; ghm5C; glucosylated hydroxymethylcytosine; m4C: *N4-*methylcytosine; m6A: *N6-*methyladenine; PT: phosphorothioation of non-bridging oxygen in DNA linkages, also called S-DNA.

+/−: at least 100-fold less activity on this substrate than on substrates with + entry.

(−), (+), based on *in vivo* restriction of phage infection or plasmid transformation with appropriate host mutant configurations; *in vitro* cleavage results have not been reported.

(+?) either m5C or m6A is recognized; these were not distinguished in the reported experiments.

−?: m6A sites tested were not cleaved, but few modified sequences were tested.

NT: not tested.

Where the name found in REBASE (and listed at the left) is not the same as that used in the cited report, the genomic locus_ID is given in the References column, or the name used in the publication.

Those modification-dependent enzymes that are classified as Type IV in REBASE (50) have been segregated (Table 1) from those classified as Type IIM (Table 2). The distinction between Type IIM and Type IV appears to reflect production of defined bands on a gel in the reported characterizations. This distinction may be misleading, as bands on a gel can result from substrate choice in some cases (see further later in the text). As no other fundamental property unites the Type IV enzymes, or distinguishes Type IIM from Type IV, these authors advocate adding Type IIM to the Type IV class.

For the most part, those functions acting on hm5C also act on m5C though with varying efficiency. EcoK Mrr, for which only *in vivo* evidence is available, may be an exception—it does not interfere with growth of hm5C-containing T-even phages. However, phage-encoded restriction inhibitors may confound interpretation of negative results obtained *in vivo* (see later in the text, ‘Phage-host arms race’).

Other Mrr-related enzymes from *Bacillus anthracis*, *Streptomyces coelicolor* and *Zymomonas mobilis* (identified bioinformatically, see later in the text) were also tested for activity *in vivo*. Transformation efficiency is reduced when a plasmid is prepared from a modifying host, compared with the same plasmid from a non-modifying host; this reduction is alleviated when the corresponding Mrr-related gene is disrupted. The specificity of this test depends on how thorough the genetic investigation was; if Dam^−^ Dcm^−^ EcoKM^+^ DNA transforms better than fully modified DNA, modification specificity could be either m6A or m5C or both, hence the question marks in the table.

The four systems listed for *S. coelicolor* 3A constitute a particularly exemplary analysis of this kind (37). In this case, all four candidate R-M systems were deleted individually and together so that the effect of each could be tested, and each system was established in the related non-restricting host *Streptomyces lividans.* For ScoA3Mrr, the effect of removing modifiable sites from the test plasmid was also examined (for M.EcoKI).

#### Diversity of functional organization

Unlike the classic Type IIP enzymes such as EcoRI and BamHI, in which catalytic residues are embedded within sequence-recognition structural elements, the modification-dependent enzymes known so far exhibit separation of DNA binding and cleavage into different domains on the same protein, or even into different polypeptide chains (Table 3 and 4). In this they resemble Type I, Type IIS or Type III enzymes, modification-blocked enzymes that also separate recognition and cleavage. For those also, multiple evolutionary events apparently have occurred to connect nuclease domains to recognition moieties (81).

**Table 3.** Characteristics of Type IV restriction enzymes

Protein	Subunits/ domains	DNA Binding	Endonuclease domain	NTP hydrolysis	Recognition site^a^, comment and references
EcoKMcrA				–	*In vitro* cleavage not reported
	N-terminal	DBD			(Y > R)m5CGR bound (55,56); hm5C/m5C discrimination (57)
	C-terminal		H-N-Hc		Bioinformatic ID (58); required for damage to DNA *in vivo* (57)
ScoA3McrA				–	Some CmCWGG and some S-DNA (PT modified) sites are cleaved (38)
	N-terminal	DBD?			Not related to EcoMcrA (38)
	C-terminal		H-N-Hc		37% identical to EcoMcrA (38)
EcoKMcrBC				GTP	Rm5C(N30-35|)-(N30-3000)-Rm5C^b^
	McrB-N	DUF3578			McrB binds DNA (40) via its N-terminal domain (59), by extrahelical modified base (60)
	McrB-C			P-loop NTPase	(61,62,63)
	McrC		PD-(D/E)XK?		Bioinformatic ID (64); Required for cleavage (39,65)
EcoKMrr				–	m6A or m5C; sequence specificity ambiguous (42,43,66)
	N-terminal	Mrr-N			Presumed DNA binding (67)
	C-terminal		Mrr-cat (D/E).. (D/E/Q) × K		Bioinformatic ID (68,69,70)
SauUS1				ATP or dATP	Sm5CNGS; two copies required for cleavage (45)
	N-terminal		PLDc-2		(45)
	Middle			P-loop NTPase	(45)
	C-terminal	DUF3427			(45); DBD?
PvuRts1I			PD-(D/E)XK?	–	mC(N11-13/N9-10|)G 2-base extensions (47) Bioinformatic ID (64)
EcoCTGmrSD		?	?	UTP>>GTP, CTP	Cuts T-even DNA (49)
	GmrS		Motifs suggested	DUF262	(71);To be confirmed
	GmrD		DUF1524		To be determined

^a^Recognition sites (represented 5′→3′) are those determined *in vitro* by binding or cleavage experiments.

^b^McrBC cleavage results in a double-strand cut near one Rm5C site (72,73,74) but requires cooperation of two sites (39,40) or a translocation block (73). The sites may be on different daughters across a fork (75). These are separated by 30–3000 (39,72,74) and may be on either strand (39,76); disposition of opposing nicks is not tightly constrained (73), and minor cleavage clusters are found ∼40, ∼50 and ∼60 nt from the m5C (74).

Degeneracy abbreviations: **B** = C or G or T; **D** = A or G or T; **H** = A or C or T; **K** = G or T; **M** = A or C; **N** = A or C or G or T; **R** = A or G; **S** = C or G; **V** = A or C or G; **W** = A or T; **Y** = C or T.

Cleavage positions are listed as (N# to top cut/# to bottom cut|). If no number is listed, the position of cleavage is not determined. Space between numbers (e.g. PvuRts1I N11-13/N9-10) indicates the range of positions at which cleavage may occur.

**Table 4.** Characteristics of other modification-dependent enzymes (Type IIM)

Protein	Subunits/ domains	DNA Binding	Endonuclease Domain	Recognition site	Comment and references
DpnI family				G m6A|TC	13 characterized isoschizomers
	N-terminal		PD..(D/E)XK		(50,77,78,79)
	C-terminal	wHTH		R m6A|TC	(78)
MspJI family				mC with preferences	Second copy stimulates cleavage
MspJI				5 mCNNR(N9/13|)	(51)
	N-terminal	SRA-like			(80)
	C-terminal		Mrr-cat (D/E)..(D/E/Q)XK		(51)
FspEI	Like MspJI			C m5C(N12/16|)	(52)
LpnPI	Like MspJI			C m5CDG(N10/14|)	(52)
AspBHI	Like MspJI			YS m5CNS(N8/12|)	(52)
RlaI	Like MspJI			V m5CW	(52)
SgrTI	Like MspJI			C m5CDS(N10/14|)	(52)
SgeI	Like MspJI			m5CNNR(N9/13|)	49% identical to MspJI; (53)
No family assigned					Information from http://www.sibenzyme.com/products/m2_type
AoxI				|RG m5CY	
BisI				G m5C|NGC	
BlsI				RYN|R Y	At least two m5C required
GlaI				R m5C|GY	(54)
GluI				GmC|NG m5C	
KroI				G| m5CCGGC	
MteI				GmCG m5C|NGm5CGm5C	
PcsI				m5CG(N5|N2)m5CG	
PkrI				Gm5CN|G m5C	At least 3 m5C required

#### Nuclease domains

Enzymes that recognize modified DNA with minimal sequence selectivity have emerged at least six times, as exemplified by the McrA, McrBC, SauUSI, Mrr, PvuRts1I and GmrSD families. These exemplars are discussed in more detail later in the text. In brief, nuclease domains have been attached covalently or (for McrC) via protein–protein interaction to domains with DNA binding and regulatory functions.

**Figure 2.**
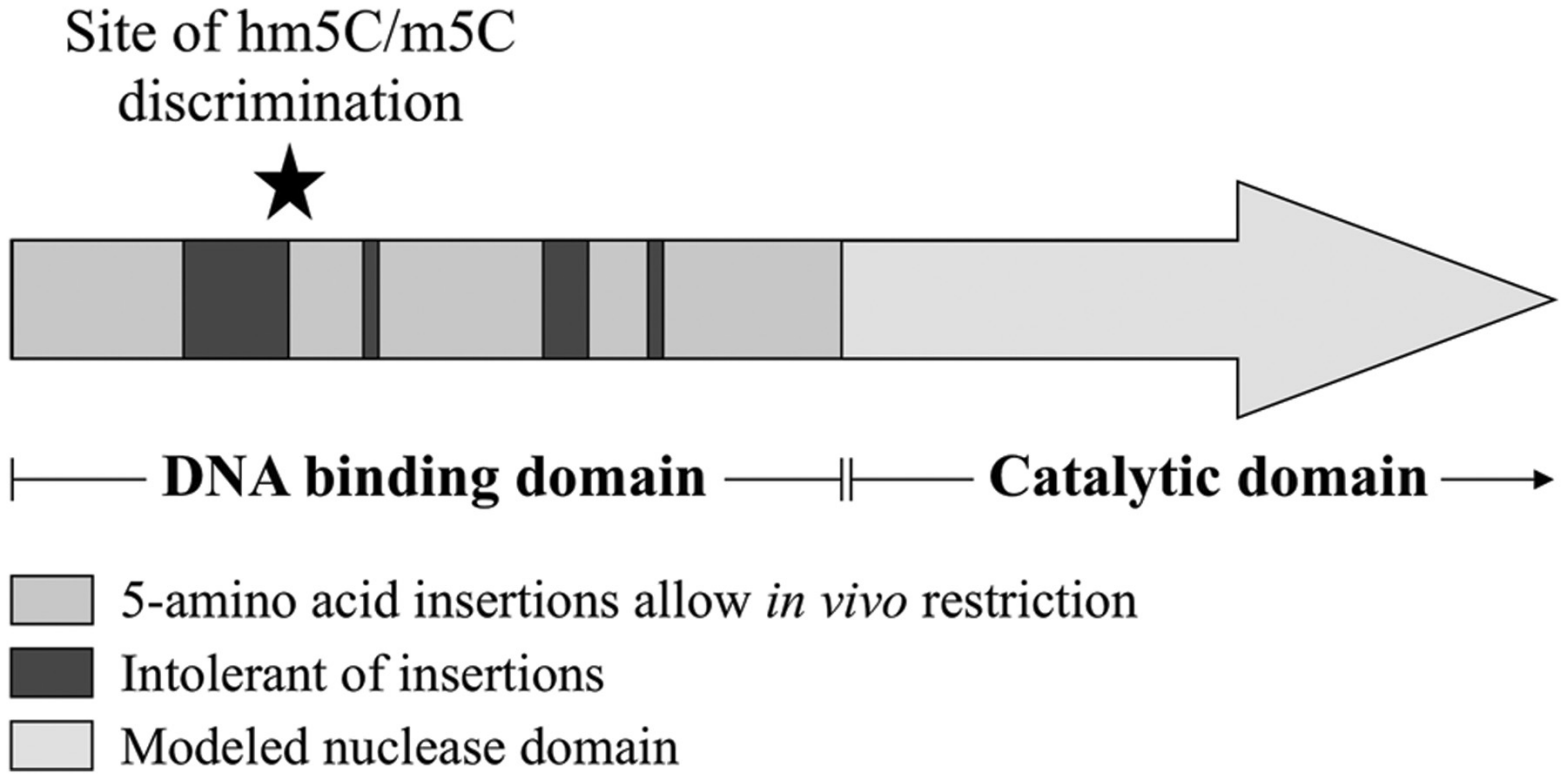
McrA functional domains. Domain function was inferred indirectly from genetic analysis by Anton & Raleigh (2004) (57). Many mutations in the N-terminal domain spared some activity in one or more of three functional tests (grey segments) while others were deficient in all activities (black segments). One mutation (asterix) was fully active on m5C-containing substrates, but fully inactive in the hm5C challenge *in vivo*. Most mutations in the C-terminus (pale grey segment) retained function in one test that was interpreted as measuring m5C binding ability. A predicted structural model by Bujnicki, Radlinska and Rychlewski (2000) (58) for this C-terminal region is compatible with these results.

EcoKMcrA carries a C-terminal H-N-Hc nuclease domain identified bioinformatically (58,82) (Figure 2). This nuclease domain is also found in modification-blocked nucleases (81). The purified binding-competent protein did not cleave under a variety of buffer conditions and cofactor additions (55). ScoA3McrA is designated ‘McrA’ due to its possession of a similar nuclease domain. For this enzyme, cleavage depends on Mn^2+^ or Co^2+^ (38) and occurs at a variable distance from PT-modified sites. Modification-blocked H-N-H REases also often exhibit unusual metal ion requirements [e.g. (83)].

**Figure 3.**
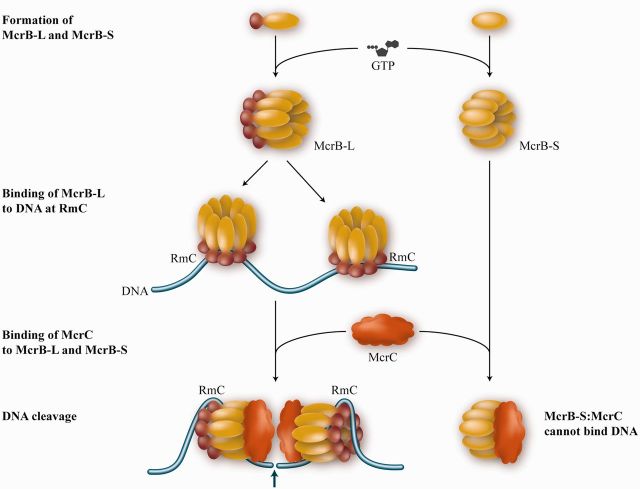
McrBC Assembly Model. Two proteins are expressed from *mcrB in vivo*. Both the complete protein (McrB-L) and a small one missing the N-terminus (McrB-S; top row) bind GTP, forming high-order multimers detected by gel filtration (second row). When visualized by scanning transmission electron microscopy, these appear as heptameric rings with a central channel. Rings of McrB-L in top views show projections that may correspond to the N-terminal DNA-binding domain (red segment). Both forms can then associate with McrC, judged again by gel filtration. McrB-L:GTP can bind to its specific substrate (RmC) in the absence of McrC (third row); in its presence, the substrate is cleaved (fourth row). GTP hydrolysis is required for cleavage (arrow): a supershifted binding complex forms in the presence of GTP-gamma-S, but no cleavage occurs. Translocation accompanies GTP hydrolysis; double-stranded cleavage requires collaboration between two complexes, or a translocation block. The path of the DNA in the figure is arbitrary, as is the conformation of McrC. Modified from Bourniquel,A.A. and Bickle,T.A. Complex restriction enzymes: NTP-driven molecular motors. Bourniquel and Bickle (84) with permission. Copyright © 2002 Elsevier Masson SAS. All rights reserved.

McrBC: The required McrC component (39,40) is the nuclease moiety (65) (Figure 3). Mutational analysis confirms that it is a PD-(D/E)XK nuclease (65), in agreement with bioinformatic classification (64). Cleavage results when McrC associates with full-length McrB:GTP complex bound to DNA and GTP is hydrolyzed (72). LlaJI, a modification-blocked restriction activity, exhibits a similar organization (85), although cleavage could not be demonstrated *in vitro*.

The classic modification-dependent enzyme DpnI also carries a PD-(D/E)XK motif (see further later in the text).

Mrr: EcoKMrr contains a variant of the PD-(D/E)XK motif (68,69) with the Mrr-N (*E. coli* K12) presumed DNA-binding domain. MspJI (see further later in the text) also carries a nuclease domain in this family. As with McrA, McrBC, and SauUSI, nuclease domain similarity does not in itself dictate modification preference properties: the single-chain R-M system LlaGI has conserved motifs characteristic of the *E. coli* Mrr protein, but this enzyme does not target methylated DNA (86).

SauUSI: This is a modification-dependent enzyme with a phosphodiesterase cleavage domain akin to one originally identified in phospholipase-D (45). Mutation of any of the four conserved catalytic residues abolishes *in vitro* activity. This cleavage domain is also found in stand-alone nucleases and modification-blocked REases (87,88). Interestingly, two of the PLDc nuclease activities have been shown to work by a transesterification reaction like that used by topoisomerases and transposases (87,89).

PvuRts1I has an apparently unusual nuclease domain [i.e. not yet identified by sources curated by the NCBI Conserved Domain Database (90)]. However, this enzyme was included in a categorization of PD-(D/E)XK families (64); a tentatively identified divalent metal ion binding site, Block B (47), corresponds to Block D of Bujnicki and Rychlewski (64). Cleavage requires Mg^2+^ ions.

EcoCTGmrSD: Functional organization is less clear but several possible nuclease motifs were identified in GmrS (71). Cleavage buffer contained Ca^2+^ and Mg^2+^ ions, and UTP.

### Sequence context recognition

Many of the modification-dependent enzymes characterized so far have little sequence specificity, in contrast to conventional modification-blocked REases. Relatively complete characterization of sequence preference and cleavage position has been carried out for Type IV enzymes EcoKMcrBC, SauUSI and PvuRts1I (Table 3) and for Type IIM DpnI and the MspJI family (Table 4). Progress has been made with binding recognition for EcoKMcrA. Cleavage conditions have been achieved for Sco3AMcrA (Table 3). For all of these, recognition of surrounding sequence context is degenerate, with preference for a neighboring base and frequently a requirement for two sites with suitable separation. DpnI is in some respects an exception, see later in the text.

The remaining nucleases in Table 4 are less well characterized. The recognition sites might form a related series. It will be interesting to learn more about the relationships among these, and how the requirement for multiple modified positions is specified, e.g. for BlsI and PkrI.

#### McrA binding domains

The two ‘McrA’ enzymes are not similar in their N-termini, with homology limited to the C-terminal nuclease domain. For EcoKMcrA (Figure 2), there is good genetic evidence that base recognition lies in the N-terminus. Extensive mutagenesis using insertion of five-amino acid linkers and classification with three functional tests allowed assignment of DNA recognition to the N-terminal portion, with the C-terminal H-N-H domain implicated in cleavage. Of particular note, a mutation discriminated *in vivo* between hm5C and m5C was found in the N-terminal domain (57). The mutant was able to fully restrict bacteriophage lambda modified by M.HpaII, but not at all phage T4 containing hm5C. *In vitro*, modification-dependent binding was achieved with the full-length His-tagged protein (55,56), yielding a putative recognition site (Y > R)mCG. This recognition site is compatible with *in vivo* observations (3,91).

Presumably, the N-terminus of Sco3A McrA also recognizes the DNA. Recognition of both m5C and the phosphorothioate (PT) moiety must be accommodated in the final reaction. As either modification is sufficient to elicit cleavage, more than one domain could be involved. Cleavage occurred near some but not all Dcm-modified sites (Cm5CWGG). Both synthetic PT-containing oligonucleotides and unmethylated PT-modified plasmid were also cleaved on both sides of a symmetrically modified site. PT modification is thought to be sequence-specific (26,29), but the details are not yet clear.

#### Novel McrB binding domain

DNA-binding resides in the McrB N-terminal domain, (40,59,60), whereas translocation and cleavage coordination reside in the C-terminal AAA+ regulatory and translocation domain (61–63,72,73). The complete translation product of *mcrB*, McrB-L, binds DNA specifically (40,72) via its N-terminal 161 amino acids (aa) (59). The crystal structure of this domain (McrB-N) in complex with DNA has recently been published (60).

McrB-N uses a strategy first discovered for DNA-MTase action (92): it flips the C base out of the DNA helix into a binding pocket for inspection. The pocket is large enough to accommodate C, m5C, hm5C or m4C, but too small if a glucose moiety is attached. Conserved residues Y64 and L68 were noted to make van der Waals contact with the methyl group of the flipped out m5C; these contacts are missing when the pocket contains C.

**Figure 4.**
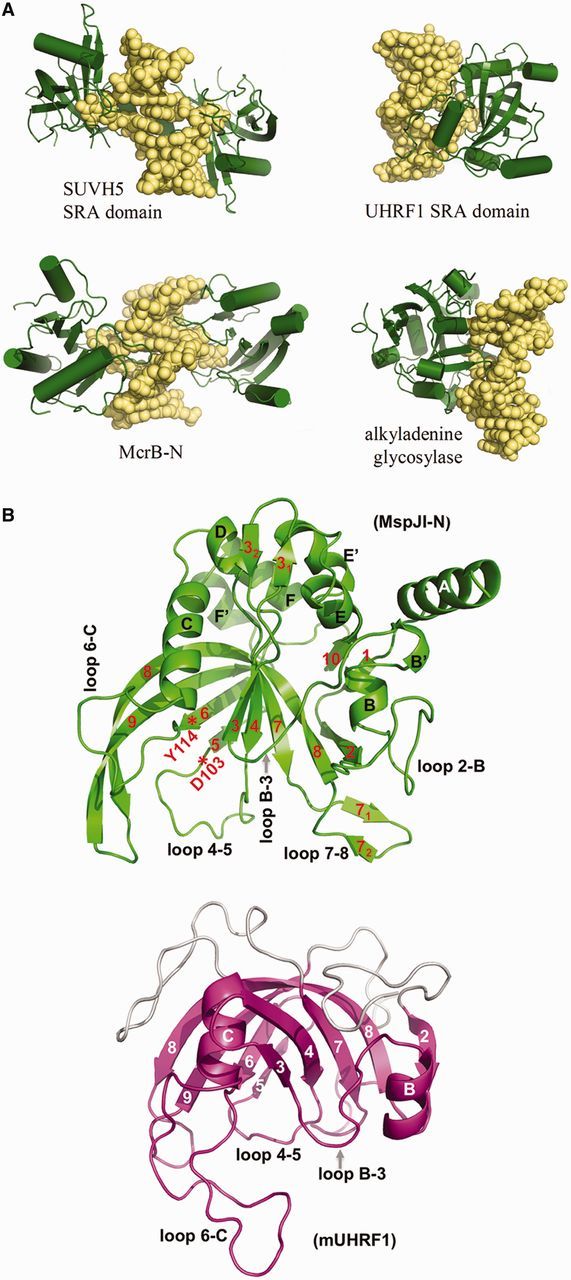
McrB-N in comparison to other base-flipping proteins. (**A**) SRA domains SUVH5 (3Q0C) and UHRF1 (2ZKD) use loops extending from a crescent formed from two beta sheets to flip C or m5C from undeformed B-form DNA into a pocket (top row), whereas McrB-N (3SSC; bottom row) uses loops from one beta-sheet to distort the DNA and flip the base. It resembles the human alkyladenine glycosylase (1BNK) (bottom row) in bending the DNA toward the major groove, while flipping the base via the minor groove. Figure 5 of Sukackaite *et al.* (60). (**B**) The SRA-like hemi-methylated 5mC recognition domains. A ribbon model of the N-terminal domain of the MspJI structure (4F0Q and 4F0P; left) compared with the SRA domain of URHF1 (PDB 3FDE; right). The crescent shape formed by interacting beta sheets and helices αB and αC are the conserved features of the SRA domain highlighted here. Loops on the concave side of UHRF1 participate in flipping the base, and similar loops presumably do so for MspJI. Two of these vary in length among family members and may play roles in sequence context specificity. Figure 2a and b from Horton *et al.* (80).

The flipping action can be compared with, but is distinct from that of, eukaryotic m5C-specific regulatory proteins that use the SET and Ring-finger-Associated (SRA) domain to read DNA modification state (Figure 4A). This domain is found in most eukaryotes, in accessory proteins (e.g. UHRF1/NP95/SUVH5) of the DNMT1 maintenance MTase, (93–95). Despite the similar strategy, the McrB-N domain is not homologous but displays a distinct protein fold (60). Binding is accomplished from the minor groove, and extraction of the C creates a 30° bend toward the major groove, resembling a glycosylase in this respect (Figure 4A). The eukaryotic proteins form a crescent from which loops project to wrap around the DNA, with recognition mediated through both major and minor grooves (94). For McrB-N, the authors suggest that the purine preference in the 5′ position might result from flexibility constraints or interaction with a non-conserved aa that occupies space left by the flipped base. Substitutions of this aa (Y41A or Y41Q) compromised binding activity.

#### Sequence specificity, novel phenotype and structural model of Mrr

In 1987, Heitman and Model discovered Mrr when they found that transfer of various foreign m6A MTases induced an SOS response due to DNA damage (42). This response to the presence of an incompatible MTase remains the principal evidence that the *E. coli* K12 Mrr protein cleaves DNA. Related proteins discussed later in the text (Type IIM) have been more tractable for *in vitro* work. No concise description of the Mrr recognition sequence has been forthcoming, although several studies have examined the spectrum of incompatible MTases (43,66,96,97). Both adenine and cytosine MTases confer sensitivity.

Mrr is also responsible for DNA damage that does not depend on methylation at all, foreign or otherwise. High hydrostatic pressure (HP) induces the SOS DNA damage response and lethality (98). The response did not depend on the activity of the endogenous MTases of *E. coli* K12 but did depend on both the presence of wild-type *mrr* and the integrity of the SOS signal generation pathway. Possibly, HP elicits a non-enzymatic modification or a structural change in DNA helicity that is acted on by Mrr. This HP phenotype was used to characterize *mrr* mutants, which were fitted into a computer-assisted model of the Mrr protein (67). An N-terminal DNA-binding winged helix was proposed, with a C-terminal nuclease domain previously identified (69). The functional importance of several conserved residues was confirmed. Several of the selected mutants with null phenotypes were isolated in a region far from the active site or binding surface identified bioinformatically. These could affect interaction with a component of the HP response. This intriguing collection of informative mutants will illuminate *in vitro* characterization.

#### Type IIM binding domains

Type IIM enzymes of two families are well-characterized with respect to cleavage (Table 4). Crystal structures for both have recently appeared.

#### DpnI: winged-helix DNA recognition

Unusually for modification-dependent enzymes, DpnI cleaves a four-base site (Gm6ATC) with high fidelity (77,99) to leave blunt ends when both strands of the site are methylated. At low concentration, the enzyme nicks the modified strand of a hemimethylated site (100). The behavior of the enzyme with respect to modification patterns within the canonical GATC site—modification of C or A, one strand or both—has been thoroughly explored (50). However, only recently has cleavage of non-canonical adenine-methylated sites been examined. Siwek and co-workers (78) found evidence for considerable relaxation of specificity at the outer base. This experiment used substrates modified by a highly non-specific adenine MTase, extensive DpnI cleavage, cloning of the fragments and sequencing of the borders.

Structure determination in the presence of DNA and validation experiments (78) place this enzyme together with the other modification-dependent enzymes, in that two domains segregate the cleavage function from sequence recognition. It also separates DpnI from the others, in that the cleavage domain also possesses some modification and sequence specificity. The main recognition is accomplished by a monomeric winged-helix domain, which binds in the major groove and recognizes the modifications on both strands in the same event. The structure does not reveal a cleavage-competent complex, however, because the cleavage domain is far from the DNA. Filter-binding experiments validated the ability of the C-terminal domain to bind alone, to do so more tightly to fully methylated than to hemimethylated oligonucleotides, and to compete with the full-length enzyme, reducing cleavage by it. Expression of the N-terminal cleavage domain alone (in low yield) allowed validation of its cleavage activity. Surprisingly, this cleavage was itself dependent on modification state and sequence of the substrate. Modeling based on the structure of the blunt-end-producing Type IIP enzyme PvuII allowed prediction that the cleavage domain approaches from the minor groove. Complete understanding of double-stranded cleavage will depend on understanding the dynamic transformations that allow the cleavage domain to approach and act at the site.

#### MspJI coupling of cleavage with DNA recognition

**Figure 5.**
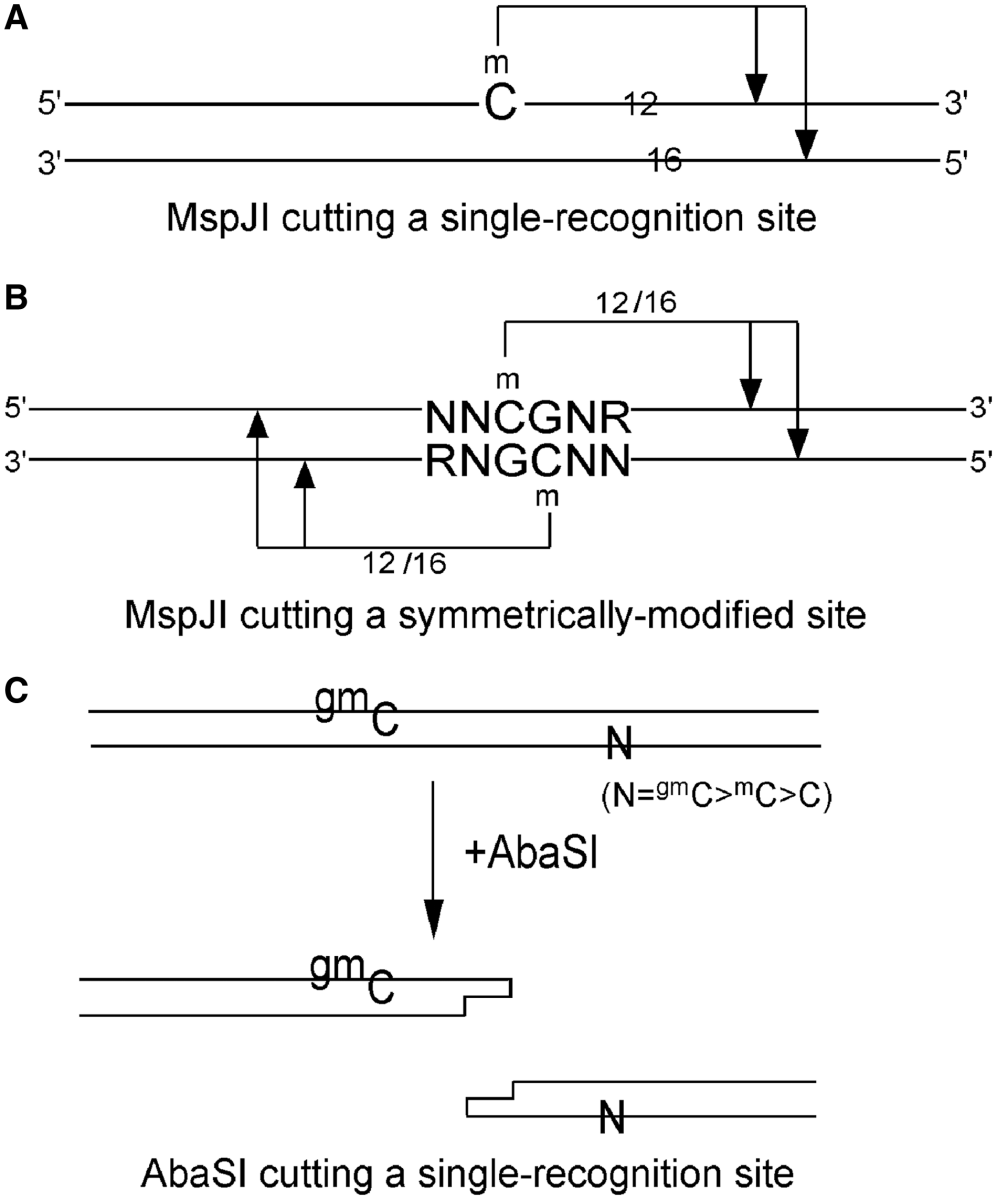
Schematic diagrams of cleavage positions for MspJI and AbaSDFI. Cleavage of both strands is elicited by a singly modified site for both MspJI (**A**) and AbaSDFI (**C**). Cleavage position is fixed relative to the modified site, but with a four-base 5′ extension for MspJI and a two-base 3′ extension for AbaSDFI. When a site is symmetrically-modified (as for CpG sites in mammalian DNA), a 32 base-pair fragment is excised from the DNA (**B**). (A) Figure 2a and (B) Figure 3a reprinted with permission from Cohen-Karni *et al.* (52). (C) Figure 5a from Wang *et al.* (47).

The six members of the MspJI family use the Mrr-cat version of the PD-(D/E)XK nuclease to cut at defined locations to one side relative to the modified base (12 bases on the modified strand, 16–17 on the other; Figure 5A); only one modified base is required for double-strand cleavage to occur (unlike McrBC) (51,52). However, these enzymes are stimulated by the presence of a second site in *cis* or in *trans*. Symmetrically modified sites (such as m5CpG:m5CpG in mammalian DNA) yield prominent bands of defined size (∼32 bp) containing a mixed population of sequences each with a m5C in the middle (Figure 5B). This behavior is recapitulated by the PvuRts1I group of enzymes (exemplified by AbaSDFI in Figure 5C), except that the distances are shorter and recognition of modification state is less well understood.

During the characterization of MspJI, Dcm (Cm5CWGG) sites were the first recognized substrate, yielding a clear banding pattern (51). Cleavage of differently modified plasmids and designed oligonucleotide substrates allowed a good assessment of both modification and sequence specificity. This family shows preference for particular bases nearby, similar to McrBC.

#### MspJI DNA recognition is mediated by an SRA-like domain

Recently, the crystal structure of MspJI without DNA has been resolved at 2.05 Å (80). Search of the Molecular Modeling Database at NCBI (101) using VAST (102) showed that the N-terminal domain was structurally similar to that of the eukaryotic SRA domain, with a crescent-shaped beta-sheet structure from which loops project (see Figure 4B and discussion earlier in the text, McrB). This structural homology allowed modeling of the DNA-bound structure, with a flipped m5C. The enzyme in the crystal is a tetramer, in which two monomers form a back-to-back dimer via the C-terminal regions that comprise the endonuclease. Two back-to-back dimers generate a tetrameric protein with two cleavage domains positioned (as in the Type IIP enzyme HindIII, used for modeling the C-terminal cleavage domain interaction with DNA) so that a 4-base 5′ extension would be created on cleavage of modeled DNA. Cleavage is most efficient at molar ratios that allow all four SRA-like domains to be occupied—too much enzyme prevents cleavage from occurring.

### Tracking and dimerization

#### McrBC as translocase

Bourniquel and Bickle (84) have reviewed much of the enzymology of McrBC, which will be briefly summarized here. The Raleigh, Bickle and Pingoud laboratories have contributed to the following consistent picture of the *in vitro* reaction. EcoKMcrBC cleavage results in a double-strand cut near one RmC site (72–74) but requires cooperation of two sites (39,40) or a translocation block (73). The sites may be on different daughters across a fork (75). These are separated by 30–3000 bp (39,72,74) and may be on either strand (39,76); cleavage occurs ∼30–35 bases from the modified base, with opposite nicks not tightly constrained (73), and minor cleavage clusters are found ∼40, ∼50 and ∼60 nt from the m5C (74). hm5C DNA elicits cleavage also (39). A ring structure is formed by 5–7 molecules of McrB in the presence of GTP (Figure 3) (103); this complex can bind to a recognition element in DNA. In the presence of McrC, translocation of the complex occurs and cleavage ensues when translocation is blocked. Collision of translocating complexes, a protein barrier or a topological barrier will elicit double-strand cleavage adjacent to one recognition element or the other. The enzyme will cleave when recognition elements are on opposite sides of a forked structure (75). This would allow action *in vivo* to prevent entry of a MTase gene even with rare sites.

Structurally, the McrB protein is proposed to be a member of the AAA+ protein family of NTPases (104), many of which form ring-shaped complexes and participate in molecular machines. ‘Sensor’ segments found in these proteins have been shown in some cases to play roles in coupling NTPase activity to intersubunit communication and movement (105). Two of three elements of the GTP-binding motif proposed by Dila *et al.* (61) were validated by mutational analysis (65,62). The third proposed motif element was identified as amino acids NTAD by Dila *et al.* Alignment of AAA+ NTPases in (104) found this aligned with the motif designated Sensor-1 in (105). An interesting result was that mutations here unexpectedly appeared to abrogate interaction with McrC instead of changing which NTP would be productive (62). It may instead play a role in coordinating GTP binding and hydrolysis with DNA binding, interaction with McrC and cleavage.

Intracellularly, the story becomes more complex, as the *mcrB* gene encodes two products of 51 and 33 kD, McrB-L and McrB-S, the latter one starting from an in-frame internal translation start site (106). Both *in vivo* and *in vitro*, McrB-S can interfere with the function of McrB-L, at least in part by forming complexes with McrC unable to bind DNA (107). Both species can form multimeric rings in the presence of GTP (103), as is usual for AAA+ NTPases (104).

#### SauUSI requires two sites and ATP hydrolysis

SauUSI was originally annotated as a putative helicase from *Staphylococcus aureus* sp. A single polypeptide is sufficient for activity both *in vitro* and also *in vivo* as a clone in *E. coli*, using modified phage λ as a challenge. The amino acid sequence contains a PLDc domain at the N-terminus. This contains a phosphodiesterase motif originally identified in Phospholipase D (108); it was validated by mutagenesis of four catalytic residue candidates. In the middle, ATPase and helicase motifs were proposed to account for ATP dependence of cleavage activity. A Domain of Unknown Function was identified at the C-terminus (Pfam DUF3427) (108) and was proposed to recognize the substrate (45).

The purified enzyme cleaves modified DNA containing m5C and hm5C but not m4C in the presence of ATP or dATP but not other nucleotides. The negative result for m4C is firm: plasmids modified at the same site by an m5C MTase (Dcm) or an m4C MTase (M.PspGI) were tested. The former (Cm5CWGG) was sensitive, whereas the latter was resistant. Thus, the sequence preference is likely to be satisfied. m6A is likely not a substrate, but few m6A-containing sites were examined.

Like McrBC, SauUSI requires the presence of two sites for efficient cleavage. Presumably, the ATPase activity participates in monitoring the presence of two sites, as for other nucleotide-dependent REases, including McrBC. The mechanism of communication is unknown. The enzyme belongs to a family of highly similar orthologues found in other sequenced *Staphylococci* (Tables 1 and 2), and more distant homologues can be found in sequenced bacterial and archaeal genomes.

## EVOLUTIONARY PRESSURES ON R-M SYSTEMS

### Evolution by selfish propagation

One way to understand the massive variety of restriction systems, and their sporadic distribution, is to locate the evolutionary drivers of enzyme diversification in the enzyme genes themselves, as selfish elements. Work from the Kobayashi laboratory has elaborated clear examples of selfish behavior in some Type II enzyme systems (109,110), in which the host becomes ‘addicted’ to the R-M system. Once a cell has acquired an R-M pair, loss of the genes results in death of that cell’s descendants, as the REase is frequently still present and able to act on the genome following loss of methylation activity. In this perspective, the role played by modification-dependent enzymes is host defense, to exclude systems with ‘foreign’ MTase patterns, and prevent the cell from loading up with parasites. The exclusion event is accompanied by the death of the cell (111–113). Weak sequence specificity of Type IV enzymes could then result from the need to control entry of a wide variety of invading systems.

The selfish aspect certainly plays a role in R-M population biology, but cannot be the whole story. Type II R-M systems can still be lost, by inactivation of the R gene first. Moreover, Type I systems escape this scenario with complex control of cleavage activity: the restriction assembly includes a methylation assembly to begin with; therefore, the R protein cannot act unless the MTase is present; in addition, failure of the methylation activity in an intact complex leads to abrogation of R activity, sometimes by action of the ClpXP protease specifically on the R protein (114–116).

Furthermore, in population terms, a cell that acquired and became addicted to an R-M system should lose in competition with a sibling that never received the system. Two factors could counter this. First, acquisition could be accompanied by an increase in the total number of copies of the R-M system in the population, as proposed for invading transposable elements. This overreplication results in more copies of the system created than are lost, whether to suicide or to other selective disadvantage [see e.g. (117,118)]. R-M gene amplification within a cell has been reported experimentally (119) but spread in a population has not been demonstrated yet. A second factor that could counter the disability of addiction is localization of competition. In a structured environment (colonies on a plate or biofilm on large or small surfaces), killing of segregants preserves limiting nutrients for lineages that retain the toxin/antitoxin pair (120,121). Much of the real world is structured, so this is an important condition

### Evolution by phage-host arms race

A second perspective supposes that the modification-dependent Type IV enzymes arose from the competitive coevolutionary interaction between phages and their hosts. This was first enunciated by Revel and Luria (2) and most recently by Black and coworkers (122); see also (123). That is, hosts used modification-blocked restriction to defend against phage infection; T-even phages developed methods of substituting modified bases for the ordinary ones; hosts developed Type IV enzymes in defense; phages added sugar or other modifications (19) to thwart Type IV enzymes; hosts extended Type IV enzymes to accommodate these decorations; finally, phages developed protein inhibitors specific for these enzymes as well. T4 phages deliver a protein inhibitor (IPI*) along with the DNA on infection, which allows growth in the presence of EcoCTGmrSD. The locus responsible for this inhibitor is highly variable among relatives of T4, as *gmrSD* is in enteric bacteria (both in distribution and in aa sequence). When phage with different IP1 alleles were tested for protection from cloned EcoCTGmrSD and its homolog EcoUTGmrSD, specificity was evident: one or the other or both or neither of the two homologs was counteracted in individual cases (122). This variability of the outcome supports the idea that phage-host interaction drives at least some of these developments.

In this perspective, the weak sequence selectivity of the Type IV systems might simply reflect the lack of endogenous targets for the enzyme. As the host does not present any hm5C and the phage is completely substituted, selection for sequence-specificity is weak. Selection would act to spare any co-resident MTases. This differs from Type II enzymes, where the MTase and REase must co-evolve to allow the host to survive. Each Type IV system is compatible with some suite of Type I-III MTases (and thus the R-M systems as a whole). Methylated or hydroxymethylated bases may not be recognized at all (EcoCTGmrSD), or the system may require one specific base in addition to the modified one (McrA, McrBC, MspJI and PvuRts1I). MTases modifying sites not including that base are then compatible, as Dcm (CmCWGG) is compatible with the McrA, McrBC and Mrr systems in *E. coli* K12.

Type IV systems that restrict methylated bases in a weakly specified sequence context confer an additional advantage in competition with phages. Many phages, such as λ, have not evolved the nucleotide-substitution strategy used by the T-even phages. These phages normally carry the modification pattern of the most recent host; if the last host expressed an MTase creating a susceptible site, the Type IV enzyme of the new host will destroy the invader and limit the infection. This may be accompanied by the death of the individual infected; therefore, protection can be conferred on the sibling population (111).

A further implication of this scenario considers the fate of a population invaded by phage. Phage survival of restriction occurs at biologically relevant frequencies (10^−^^6^-10^−^^2^). The survivors of restriction carry the particular methylation pattern of the particular cell and thus are resistant to all restriction systems it might have carried (Types I–IV). A bacterial population as a whole then benefits from mechanisms that diversify the suite of R-M systems so that such surviving phage do not have free access to the entire population. The extreme variability of R-M system content in isolates of the same species is compatible with this idea [see e.g. (124); REBASE Genomes http://tools.neb.com/∼vincze/genomes/]. Such variability also limits and shapes interstrain gene transfer (115,125,126).

The Raleigh laboratory has built on elegant genetic work with Type I enzymes in the Murray laboratory (127–130) to investigate a locus designated the ‘Immigration Control locus’, or ICR, that exemplifies variable R-M content. Alternative DNA segments containing R-M systems are located at the same defined location in most *E. coli* chromosomes between the *yjiS* and *yjiA* genes. The ICR in the non-restricting *E. coli* C strain [used in the original definition of the R-M phenomenon (131)] contains a remnant of a Type I enzyme R gene and is 13 kilobase shorter than the same region in *E. coli* K12 (132). The mechanism of segment replacement is still unknown. The ICR would be an example of the ‘defense islands’ analyzed by the Koonin group (133). ‘Defense islands’ contain genes that can defend against phage or other invading DNA; these exhibit bioinformatic properties similar to ‘mobilome islands’ containing mobilization genes (transposases for example). However, the mechanism of mobilization has not been identified for the ICR.

## FINAL THOUGHTS

The extreme diversity of R-M systems that recognize ordinary DNA seems likely to be approached by the diversity of Type IV restriction systems. Type IV enzymes are hard to find, as most detection methods depend on development of genetic systems for each taxon, or on serendipity. Those characterized so far mostly stem from initial genetic investigation of limits on infection, transformation or transduction. Barriers encountered provide leads to the genes involved. Bioinformatic analysis has helped to identify relatives, which may be more tractable to biochemical investigation than the example originally found. This approach has pitfalls: the gene encoding MspJI was first thought to code for an enzyme recognizing an unmodified site because it is immediately adjacent to an (inactive, it is now thought) cytosine MTase gene. Providentially, the first expression host was devoid of sensitive sites, whereas the first test substrate contained some (51). A combination of biological experiments with bioinformatics and biochemistry will be needed to reveal the full spectrum of Type IV enzymes that may lurk within the vast universe of unidentified ORFs in bacterial systems. One might begin with those strains whose genomes carry few Type II systems: *Bacillus*, or *Corynebacterium*, as opposed to *Helicobacter* or *Neisseria* [see the Genomes section of REBASE (50)].

The role of ‘defense islands’ and their relation to the ‘mobilome’ in bacterial population biology remains to be determined. If a defense island is similar to a mobilome island, there should be a mechanism of mobilization nearby, which would boost the contribution of ‘overreplication’ to the account of selections acting on R-M systems. R-M systems of all types can be found on or adjacent to known mobilizing elements (134,135), but have not been shown to move experimentally.

On another note, it may turn out that evolutionarily there is a continuum between the apparently modification-dependent and modification-blocked paths. One relative of McrBC predicted by bioinformatics analyses is LlaI, a system that recognizes an unmodified sequence and requires two MTases to support it (136). The enzyme BamHI prefers to cleave DNA with m6A in its GGATCC site, and mutants can be isolated that require this modified base (137). Are there native systems similarly protected by modification of one position in the recognition site but dependent on modification at a different one? An interesting evolutionary series can be imagined.

## FUNDING

New England Biolabs (E.A.R.). Funding for open access charge: New England Biolabs (to E.A.R.).

*Conflict of interest statement*. None declared.
